# The role of mobile health applications (mHealth apps) in reshaping the body weight for better healthcare: A cross-sectional study

**DOI:** 10.12669/pjms.40.9.9064

**Published:** 2024-10

**Authors:** Moath Mohammed Albarrak, Nasriah Zakaria, Sultan Ayoub Meo

**Affiliations:** 1 Moath Mohammed Albarrak Public Health Operation Center, Ministry of Health, Riyadh, Saudi Arabia; 2 Nasriah Zakaria. Department of Computer Sciences and Information Systems, College of Applied Sciences, Al Maarefa University, Riyadh, Saudi Arabia, and E-health Unit, Faculty of Medicine, University Malaya, Malaysia; 3 Sultan Ayoub Meo Department of Physiology, College of Medicine, King Saud University, Riyadh, Saudi Arabia

**Keywords:** Obesity, Mobile health application, BMI, weight reduction, Saudi Arabia

## Abstract

**Objectives:**

Obesity is a chronic, multifaceted, leading health problem causing numerous non-communicable diseases with a huge burden on the global healthcare system and economies. Worldwide, multiple interventions have been familiarized for weight reduction strategies, however, in recent years, mobile health applications (mHealth apps) for reshaping weight reduction have been introduced. Therefore, this study aimed to investigate the role of mobile health applications in reducing weight among users in Riyadh, Saudi Arabia.

**Methods:**

This questionnaire-based cross-sectional study was conducted in the Department of Family and Community Medicine, and Medical Education, College of Medicine, King Saud University, Riyadh from May 2019 to December 2022. A total of 502 participants, 332 (66.1%) females and 170 (33.9%) males aged 18-50 years, using smartphones, residing in Riyadh, Saudi Arabia were recruited. The questionnaire was distributed via social media platforms including WhatsApp, and emails and information were recorded.

**Results:**

Among 502 participants, 221 (44.02%) were using mobile health applications and 281 (55.98%) did not use mobile health applications. The results revealed that the participants who have been using mHealth apps for the last six months had significantly decreased body weight compared to those who did not use the mHealth apps (p=0.001).

**Conclusions:**

The study participants using mobile health applications (mHealth apps) had significantly reduced their body weight and felt healthier. The mHealth apps provide health-related information regularly to individuals to track their weight, monitor diet, and physical activity, and information about their overall health.

## INTRODUCTION

Overweight and obesity are characterized by an excess accumulation of body fat which is considered a risk to human health.^1^ The prevalence of overweight among adult men and women is about 39% and 13.1% for obesity.^2^,^3^ In Saudi Arabia, the literature highlights that there has been a steep increase in the obesity rate over the years 1998-2005 and 2011.^4^ The recent reports highlight that the epidemiological estimates of overweight and obesity among men and women in Saudi Arabia continued to expand and are particularly alarming. The prevalence among adults was about 60% and children and adolescents were 20–60%.^5^

Obesity is a major health hazard, it contributes to different non-communicable diseases, especially diabetes mellitus, ischemic heart diseases, sleep-breathing disorders, and different forms of cancers.^6^ This issue contributes a huge burden on the health system as well as on the individual level in which it mandates executing well-developed strategic plans to prevent and manage overweight and obesity.^7^ By 2030, the obesity-allied complications are estimated to cost up to 66 billion per annum in the USA and around £1·9 to 2 billion per annum in the UK.^8^

The scientific literature proposed multiple interventions to reduce body weight including exercise, lifestyle modification^9^,^10^ and pharmacotherapy^11^, which showed a positive effect on weight reduction, but still, some of these interventions showed limitations requiring more financial input and time-consuming processes.^12^ With the widely distributed and continuously growing market of mobile phones, the International Telecommunications Union (ITU) reported that as of 2018, the number of mobile-cellular telephone subscriptions has exceeded the total world population; whereas in Saudi Arabia, the number of mobile-cellular telephone subscriptions has reached 40,210,965 by the end of 2017.^13^

Mobile Health uses wireless devices for monitoring health and establishing links between individuals and medical care professionals.^14^,^15^ The literature explored the impact of smart mobile phone health applications on human health and managing diseases by providing timely text-message support from a health care professional and health information.^16^-^18^ An online survey was conducted in Saudi Arabia and the authors assessed the general population’s attitude toward health information encountered on Twitter. The survey results revealed that the users were satisfied with Twitter as a source of health information, particularly regarding nutrition and weight loss.^19^ The impact of mobile health application usage on weight reduction highlights that the mobile app users who monitored their weight more frequently had more effect on weight reduction.^19^

The literature highlights the adherence of people to mobile health applications for weight loss compared to websites and paper diaries and found more feasibility towards smartphone applications.^20^ This is a cost-effective method for weight reduction when applied on a population basis, and the mHealth apps are becoming more feasible to install and use. This study aimed to investigate the role of mobile health applications’ usage in weight reduction in the Riyadh region, Saudi Arabia.

## METHODS

This questionnaire-based cross-sectional study was conducted in the Department of Family and Community Medicine, and Medical Education, College of Medicine, King Saud University, Riyadh, Saudi Arabia during the period May 2019 to December 2022.

### Inclusion and Exclusion criteria:

The inclusion criteria for participants were based on whether the participants were using mobile health applications known as (the mHealth apps users’ group), and participants who were not using mobile health applications known as (non mHealth apps users’ group). The mHealth apps user group participants were adults 18 years or older, who have had a smartphone, with a mobile health application installed for at least six months, and frequently use the mobile apps daily, living in Riyadh region were included in the study. Initially, 734 participants visited the survey link, and finally, 502 participants were eligible for inclusion criteria. However, 17 people were excluded for refusing participation, 183 responders for residing outside the Riyadh region, and 32 participants for using apps less than once per month.

### Questionnaire:

A well-established, questionnaire was prepared by the research team in an English language, translated into Arabic, and reviewed by two senior faculty members. The questionnaire was piloted among ten individuals through electronic devices (smartphones and iPads). The purpose of this pilot study was to identify questions that might not be clear to the participants, to find out questions that may need modification, or add extra options to the answer, or to ask additional questions. After that, the questionnaire was distributed via social media platforms, including WhatsApp and emails to collect the data.

### Data collection:

The questionnaire consisted of demographic data: age, gender, height, weight, current weight, and weight before six months. Marital status, nationality, educational level, place of residence, and socioeconomic status. The participants were asked to enter the information and weight-based data in the application with current and six months earlier information. Moreover, the information on app usage, the reason to use or not use, the name of the app, and frequency of usage of apps. The items in the questionnaire were adopted from Krebs et al. and Duncan et al. study.^21^ The prevalence was recorded by asking questions about the use of mobile health applications, calories, steps counters, workouts etc. The self-monitoring factors including body weight, baseline calories, physical activities, weight, and food habits for six months were recorded. To obtain the total number of data entries during the six months for each category, the total number of days was counted and recorded the required information.

### Ethical approval:

The ethical approval was obtained from the Institutional Review Board, College of Medicine, King Saud University, Riyadh, KSA (Ref # E-19-4035, dated May 15, 2019).

### Statistical Analysis:

The data were entered and analyzed through the statistical package SPSS 25 (SPSS Inc., Chicago, IL, USA). All categorical variables such as age, gender, marital status, nationality, educational level etc. were presented as numbers and percentages. Whereas continuous variables such as body weight, and BMI were expressed as Mean ± SD and median. The parametric tests were used to compare groups on normally distributed variables and non-parametric tests were used when data were skewed / non-normal data.

The Chi-square / Fisher’s exact test was used according to whether the cell expected frequency is smaller than five, and it was applied to determine the significant association between categorical variables. An independent t-test was applied to determine the mean significant differences between users and non-users of health applications and anthropometric measurement. Furthermore, the Mann-Whitney U test is used to compare differences between two independent groups’ users and non-users of health applications concerning differences in body weight. A two-tailed p-value less than 0.05 was considered statistically significant.

## RESULTS

A total of 734 participants responded to the questionnaire survey, and 502 participants met the inclusion criteria, while 17 were excluded for refusing participation, 183 responders were residing outside the Riyadh region, and 32 participants for using apps less than once per month. The majority of the participants who used health applications were young, single, and reported good general health.

As shown in Table-I, 502 participants were included in this study, their ages ranged from 18 to more than 50 years, and most of the participants were 18-25 years old. Among the respondents 66% were females and 44% were males. All the participants were Saudi (95.4%); 58.6% of respondents were single, 39.8% of them were married and 1.6% of them were divorced. one-third (39.6%) of participants had an average monthly income of 1000/ Saudi Riyals, while 20.5% had a 10000-20000 monthly income (Table-I).

**Table-I:** Basic demographic characteristics of participants (n = 502).

Characteristics	Description	N (%)
Age Group	<= 25	258 (51.4%)
	26 – 35	131 (26.1%)
	36 – 45	73 (14.5%)
	> 45	40 (8.0%)
Gender	Male	170 (33.9%)
	Female	332 (66.1%)
Marital status	Single	294 (58.6%)
	Married	200 (39.8%)
	Divorced	8 (1.6%)
Nationality	Saudi	479 (95.4%)
	Non-Saudi	23 (4.6%)
Educational level	Primary	2 (0.4%)
	Secondary	3 (0.6%)
	High school	78 (15.5%)
	Bachelor’s degree	367 (73.1%)
	Higher education	52 (10.4%)
Monthly Income	0 – 1000 SAR	199 (39.6%)
	1000 – 5000 SAR	100 (19.9%)
	10000 – 20000 SAR	103 (20.5%)
	20000 – 30000 SAR	23 (4.6%)
	5000 – 10000 SAR	58 (11.6%)
	> 30000 SAR	19 (3.8%)
In general, you would say your health is:	Poor	9 (1.8%)
	Fair	59 (11.8%)
	Average	122 (24.3%)
	Good	191 (38.0%)
	Excellent	121 (24.1%)
Health App users and non-users	User	221 (44%)
	Non-user	281 (56%)

Table-I also shows the overall health of the study participants, 24.1% of the participants’ health was excellent, 38% of respondents’ health was good, 24.3% average, 11.8% fair, and 1.8% of them had poor general health (Table-I). In this study, 221 (44%) of the participants were mHealth application users while 281 (56%) of them were nonusers Table-II. Table-III and Table-IV highlights the reasons for using health applications by the participants and the distribution of applications used.

**Table II:** Distribution of chronic disease among participants.

***Do you suffer from any chronic disease?***	
Yes	56 (11.2%)
No	432 (86.1%)
I do not know	14 (2.8%)
***Participant’s response to chronic disease***	
List of Chronic Diseases	n = 56
Cardiovascular disorders	7 (12.5%)
Endocrine disorders (like diabetes)	18 (32.1%)
Respiratory disorders	10 (17.9%)
Musculoskeletal disorders	1 (1.8%)
Neurological disorders	3 (5.4%)
Mental health disorders	1 (1.8%)

**Table III:** Distribution of causes for using health applications (n = 221).

Reason/s for using the App	N (%)
Weight loss	75 (33.93%)
How much activity I do	114 (51.55%)
Watch what I eat	14 (6.33%)
Show/teach me exercise	32 (14.44%)
Health measures heart rate, blood sugar, etc.	28 (12.66%)
Check my medical records	5 (2.26%)
Other (please mentioned)	
Healthy food and follow my intake.	1 (0.45%)
Water drinking reminder.	1 (0.45%)
Book Appointment	1 (0.45%)
Calculate fasting hours.	1 (0.45%)
Measure distances for cycling and walking.	1 (0.45%)
Follow activity.	1 (0.45%)
Follow up with nutritional practitioner.	1 (0.45%)
Share exercise with friends.	1 (0.45%)
Count my steps and ensure I increase them.	1 (0.45%)
Already installed in the phone	1 (0.45%)

**Table IV:** Distribution of applications used for the last six months or more (n=221).

Google Fit	19 (8.60%)
Apple Health App	91 (41.8%)
Samsung Health	20 (9.05%)
Mi Fit	29 (13.12%)
Huawei Health	21 (9.50%)
Home Workout – No Equipment	11 (4.98%)
Step counter –Pedometer Free & Calorie Counter	8 (3.62%)
Calorie Counter – My Fitness Pal	30 (13.57%)
Fitbit	22 (9.95%)
Polar	8 (3.62%)

Mobile health applications, Apple Health, Calories Counter, Mi Fit, and Huawei Health were the most common applications used by the participants for weight loss and follow-up activity. Table-V shows the difference between current weight and before six months in addition, it shows that people who are using health applications tend to feel healthier in general (Table-VI).

**Table V:** Anthropometric measurement of subjects/participants concerning health application users and non-users.

	Application User and Non-user		P - value

	User	Non-user	
Current BMI	25.42 ± 6.12	25.78 ± 6.87	0.537
BMI before six months	26.07 ± 6.78	25.68 ± 7.06	0.532
Current Weight (kg)	68.8 ± 17.46	69.17 ± 17.72	0.811
Weight before six months (kg)	70.66 ± 19.56	68.96 ± 18.43	0.323
***Difference between current weight and before 6 months (kg)***			
Median (IQR)	3 (5 – 1)	2 (4 – 1)	0.001

**Table VI:** Impact and association between mobile Health application users and non-users among study factors.

		Application User and Non-user		P - value

		User (n = 221)	Non-user (n = 281)	
Age Group	<= 25	126 (57.0%)	132 (47.0%)	0.025
	26 – 35	51 (23.1%)	80 (28.5%)	0.172
	36 – 45	27 (12.2%)	46 (16.4%)	0.190
	> 45	17 (7.7%)	23 (8.2%)	0.840
Gender	Male	80 (36.2%)	90 (32.0%)	0.327
	Female	141 (63.8%)	191 (68.0%)	0.327
Marital status	Single	144 (65.2%)	150 (53.4%)	0.008
	Married	75 (33.9%)	125 (44.5%)	0.017
	Divorced	2 (0.9%)	6 (2.1%)	0.275
Nationality	Saudi	213 (96.4%)	266 (94.7%)	0.361
	Non-Saudi	8 (3.6%)	15 (5.3%)	0.361
Educational level	Primary	0 (0.0%)	2 (0.7%)	0.209
	Secondary	1 (0.5%)	2 (0.7%)	0.708
	High school	37 (16.7%)	41 (14.6%)	0.509
	Bachelor’s degree	159 (71.9%)	208 (74.0%)	0.603
	Higher education	24 (10.9%)	28 (10.0%)	0.744
Monthly Income	0 – 1000 SAR	94 (42.5%)	105 (37.4%)	0.240
	1000-5000 SR	47 (21.3%)	53 (18.9%)	0.503
	10000-20000 SR	46 (20.8%)	57 (20.3%)	0.884
	20000-30000 SR	9 (4.1%)	14 (5.0%)	0.628
	5000 – 10000 SR	18 (8.1%)	40 (14.2%)	0.034
	> 30000 SR	7 (3.2%)	12 (4.3%)	0.520
BMI before six months	<18.5 (underweight)	13 (5.9%)	27 (9.6%)	0.126
	18.5 - 24.9 (Normal)	101 (45.7%)	119 (42.3%)	0.452
	25 - 29.9 Overweight	57 (25.8%)	76 (27.0%)	0.752
	> 30 Obese	50 (22.6%)	59 (21.0%)	0.661
In general, you would say about your health is:	Poor	0 (0.0%)	9 (3.2%)	0.007
	Fair	26 (11.8%)	33 (11.7%)	0.994
	Average	42 (19.0%)	80 (28.5%)	0.014
	Good	94 (42.5%)	97 (34.5%)	0.066
	Excellent	59 (26.7%)	62 (22.1%)	0.228

## DISCUSSION

This study was conducted on smartphone owners to explore the relationship between mobile health application usage and weight reduction. It was identified that the participants using mobile health applications significantly reduced their body weight and felt healthier. In agreement with the present study findings, Han et al.^22^ reported that mHealth app users who frequently monitor their health-related behaviors reduce body weight in a fleeting period. Similarly, Lugones-Sanchez et al.^23^ conducted a multicenter, randomized controlled clinical trial and showed that compared with standard counselling, adding a self-reported app and a smart band obtained beneficial results in terms of weight loss and reduction. Patel et al.^24^ suggested that using tailored weight and calorie goals and a commercial app can produce clinically significant weight loss in one-third of individuals. In the present study, Apple Health, Calories Counter, Mi Fit, and Huawei Health applications were the most common mobile health applications used by the participants for weight loss and follow-up activity. We found that participants who used health applications significantly reduced their body weight.

As for the effectiveness of mobile health applications, the users found that weight-management apps helped them lose weight compared to overweight or obese users. It was also identified that mobile apps for weight loss did not affect the weight of overweight people and were useful for individuals who were willing to self-monitor their calories.^25^ Additionally, Alnasser et al.^26^ conducted a study to investigate the impact of health apps on weight loss among 240 overweight volunteers who used the apps for four months. The results revealed that mHealth has a positive impact on body weight loss while comparing App users and non-users about weight reduction. It shows that the difference between previous and current weight in those who are using mobile health applications has reduced their body weight.

It is important to highlight that the prevalence of obesity in the Gulf region has become a serious public health issue. The GCC countries have the highest obesity rates in the world.^27^ In such situations, the use of mobile health applications may support people in reducing weight loss and to combat against overweight and obesity. The appropriate use of mobile phone health applications is important for health promotion and academic activities.^28^

### Strengths and Limitations:

The present study contributes to the existing literature that mHealth apps are simple, easily accessible, and effective tools for reducing body weight within a fleeting period. This study has some limitations. First, the limited sample size, therefore, in the future large sample-sized studies are needed to investigate the impact of mHealth apps in reducing body weight. Second, self-reported data from the participants may not be adequate. Third, it remains unclear whether the study participants may use only the app to lose body weight or other physical activities such as using the gym, personal training, or diet control therapy.

## CONCLUSIONS

It was concluded that people who are using mobile health applications (mHealth apps) reduced body weight and tend to feel healthier in general in a brief period of six months. Mobile health applications, Calories Counter, Mi Fit, and Hawaii Health were the most common applications used by the participants for weight loss and follow-up activity. However, further large sample-sized studies from various regions of the world are needed to reach better conclusions.

The mHealth apps play a significant role in weight reduction and overall health management. These apps provide regular basis information to individuals to track their weight, monitor diet, physical activity, exercise, and information about their overall health. The mHealth apps with a balanced diet, regular exercise, and professional guidance from healthcare providers can lead to more successful and sustainable weight loss outcomes. Additionally, individuals should choose mHealth apps that align with their specific goals and preferences to maximize their effectiveness.

## Authors’ contributions:

**MAA:** Study design, literature review, data collection, analysis, and manuscript writing.

**NZ:** analysis, and manuscript writing and responsible for the accuracy of the study.

**SAM:** Manuscript writing.
